# Evaluation of Cardiac Repolarization Indices in Epilepsy Patients Treated with Carbamazepine and Valproic Acid

**DOI:** 10.3390/medicina56010020

**Published:** 2020-01-06

**Authors:** Ramazan Asoğlu, Mahmut Özdemir, Nesim Aladağ, Emin Asoğlu

**Affiliations:** 1 Cardiology Department, Adıyaman University Training and Research Hospital, 02000 Adıyaman, Turkey; 2 Cardiology Department, Bayrampasa Kolan Hospital, 34000 İstanbul, Turkey; dr.mahmutozdemr56@yahoo.com; 3 Cardiology Department, Van Training and Research Hospital, 65000 Van, Turkey; nesimaladag@hotmail.com; 4 Cardiology Department, Mardin Community Hospital, 47000 Mardin, Turkey; asemctf@hotmail.com

**Keywords:** ECG, epilepsy, cardiac repolarization, carbamazepine, valproic acid

## Abstract

*Background and Objectives*: Epilepsy patients have a higher risk of sudden unexplained death compared to the rest of the population. Cardiac repolarization abnormalities might be seen in epilepsy during interictal periods. We aimed to evaluate the changes in electrocardiography (ECG) parameters in generalized tonic-clonic seizure patients treated with carbamazepine or valproic acid (VPA) drug. *Materials and Methods*: A totally of 129 subjects (66 epilepsy patients, 63 healthy subjects) were enrolled in the study. Of the patients, 36 were on carbamazepine and 30 were on VPA. There were 12-lead ECGs obtained from all participants. RR interval (time between consecutive R peaks), QT interval (defines the period of ventricular repolarization), corrected QT (QT interval corrected for heart rate; QTc), QTc-maximum (QTc-max), QTc-minimum (QTc-min), QTc dispersion (QTcd), P (atrial depolarization )-maximum (P-max), P-minimum (P-min) and P dispersion (Pd) were measured. *Results*: QTd (QT dispersion), QTcd, and Pd values were significantly higher in the patients compared to the controls (*p* < 0.01). QTcd, Pd, and P-max values were statistically higher in male patients compared to healthy male controls. QTcd values were significantly higher in female patients using carbamazepine compared to the female patients on VPA and healthy controls (*p* = 0.01). Male patients using VPA had significantly higher QTcd values against the male population in carbamazepine and control groups. *Conclusions*: This study demonstrated that QTd, QTcd, and Pd values were significantly higher in epilepsy patients than in healthy controls. In addition, female patients using carbamazepine and male patients using VPA were prone to ventricular arrhythmia compared to the control group.

## 1. Introduction

Epilepsy, a chronic disorder of the central nervous system, includes recurrent seizures or unusual behavior, and sometimes loss of consciousness [1]. The risk of Sudden Unexpected Death in Epilepsy (SUDEP) is an important concern for patients with epilepsy that causes an unexpected, sudden, nontraumatic death of a person who is reasonably healthy. Epilepsy patients have an increased risk of sudden unexplained death, SUDEP being the most dangerous complication of epilepsy [2]. SUDEP usually occurs without any apparent reason except status epilepticus (SE). Generalized tonic-clonic seizure (GTCS) is the leading risk factor for SUDEP [3]. It has been reported that postmortem examinations for SE have not revealed any toxicological or structural reasons [4]. The population-based studies have estimated SUDEP incidence between 2–10 per 1000 person/years [2]. SUDEP is a multifactorial disease and its mechanism is complicated. A variety of factors, such as anti-epileptic medications, autonomic dysfunction, heart rate variability, and cardiac arrhythmias, contribute to SUDEP development during and between seizures [5]. 

It is not uncommon to observe cardiac rhythm disorders during ictal, interictal, and postictal phases in patients with epilepsy. Cardiac repolarization abnormalities (prolonged or shortened QT intervals (QT interval defines the period of ventricular repolarization), increased QT dispersion (QTd) might be seen in epilepsy during interictal periods [6]. Shorter QT intervals were demonstrated in epilepsy patients previously [7]. On the contrary, Drake et al. could not show any differences in QT intervals between epilepsy patients and healthy individuals [6]. Alterations in ventricular conduction which is related to uncontrolled epilepsy might result in cardiac dysfunction [8]. 

Antiepileptic drugs (AEDs) have different effects on the cardiovascular conduction system. Use of carbamazepine may cause atrioventricular blocks, bradycardia, and arrhythmias [9]. Moreover, carbamazepine is one of the main reasons for the QT prolongation in epilepsy patients [10]. Valproic acid (VPA) is an AED which is used to treat several types of seizures in adults and children [11]. A recent study has shown longer QT intervals in epilepsy patients treated with VPA compared to healthy subjects [12]. On the other hand, Amin et al. showed that the heart rate and the total number of atrial ectopics were reduced after VPA administration in patients with GTCS [13]. It is known that carbamazepine and VPA alter cardiac conduction in epilepsy patients. In this study, our aim was to evaluate the changes in electrocardiography (ECG) parameters, including corrected QT dispersion (QTcd) and P (atrial depolarization)-wave dispersion (Pd), in patients with GTCS who were on carbamazepine or VPA. 

## 2. Materials and Methods

This study was planned as a cross-sectional study and it was conducted between May 2014 and June 2015 in the neurology and cardiology departments of Van Training and Research Hospital. The study population included 66 patients and 63 healthy controls. Sixty-six patients diagnosed with GTCS and epilepsy according to the International League Against Epilepsy (ILAE) criteria [14] were enrolled. Of these 66 patients, 36 were on carbamazepine and 30 were on VPA. The clinical history of the patients and GTCS semiology together with electroencephalography (EEG), brain magnetic resonance imaging (MRI), or brain computed tomography (CT) were used for diagnostics. Age, gender, and body mass index (BMI) matched 63 healthy controls enrolled to the study. 

All patients underwent detailed history taking, cardiac and neurological examinations, routine biochemistry, electrocardiography (ECG), and transthoracic echocardiography (TTE). The inclusion criteria were: Being diagnosed with GTCS and to be aged between 18–65 years old. The exclusion criteria were: Being on medications other than carbamazepine and VPA that could affect ECG intervals (e.g. antibiotics, anti-arrhythmias, digitalis, antihistamines, tricyclic antidepressants, central nervous system (CNS) stimulants, and lithium), using a cardiac pacemaker, having cardiac rhythm disorders, diabetes, bundle branch block, or known cardiac and psychiatric disease, liver failure, substance abuse, acute or chronic infection, and serum electrolyte abnormalities.

The patients were rested for 15 minutes to stabilize the heart rate and the traces of 12-lead ECGs were recorded. The Nihon Kohden Cardiofax GEN ® device (Nihon Kohden, Tokyo, Japan) was used with a rate of 25 mm/s in 12-channeled form and with a standard of 10 mm/mV. A magnifying glass was used for measurements. The ECGs were examined by two different cardiologists, who did not have any information about the study, and they did not have any contact during the interpretation of the results. The RR interval was defined as the time intervals between two consecutive heartbeats (R-waves). QT interval (distance between the start of QRS (ventricular depolarization) and the end of the T-wave (ventricular repolarization)) was measured and minimum (QT-min) and maximum QT (QT-max) values were recorded. The end of the T-wave was defined as the intersection of the terminal limb of the T-wave with the isoelectric baseline. The Bazett formula (QTc = QT/√RR) (QT interval corrected for heart rate; QTc) was used to assess the corrected QT (QTc); hence, QTc-minimum (QTc-min), the QTc-maximum (QTc-max), and QTc dispersion (QTcd) values were recorded [15]. QTcd values >50 ms were considered as pathologic. The intra-observer and inter-observer variability on QTcd (0.94 and 0.92) and Pd (0.88 and 0.90) were excellent. In all derivations, P-wave duration was checked, and Pd (difference between the maximum and minimum P-wave duration) was determined using the following formula: Pd = (Pmax) − (Pmin). Blood pressures and heart rates were recorded.

A complete TTE was performed in all participants following the American Society of Echocardiography guidelines. M-mode from the parasternal long-axis view was used to determine the left ventricular end-systolic and end-diastolic diameters (LVESD, LVEDD). Pulse wave Doppler echocardiography measured the early and late diastolic mitral flow velocities (mitral E and mitral A, respectively). The thicknesses of posterior wall (PW), left atrium diameter, inter-ventricular septum (IVS), ejection fraction (EF), and aorta systolic and diastolic diameters were measured. 

All participants gave their written informed consent before the study. This study was performed under the principles stated in the Declaration of Helsinki. The local Ethics Committee of the hospital approved the study (086 – 26 September 2014).

### Statistical Analysis

SPSS (IBM Corp. Released 2011. IBM SPSS Statistics for Windows, Version 20.0. Armonk, NY: IBM Corp.) was used for data analysis. The Kolmogorov–Smirnov test was used assess the distribution of the variables. Normally distributed variables were expressed as mean ± standard deviation (SD). The categorical variables are given as percentages. Student’s unpaired t-test or the Mann–Whitney U test were used to assess differences between two groups for parameteric and nonparametric variables, respectively. Fisher’s exact or chi-square tests were used to calculate the frequencies of nominal variables. For multiple comparisons, one-way analysis of variance (ANOVA) test followed by the Tukey post hoc test was used, and *p* < 0.05 was taken as statistical significance.

## 3. Results

The cardiologic assessment results and demographic data of the participants are presented in Table 1.

**Figure 1:**
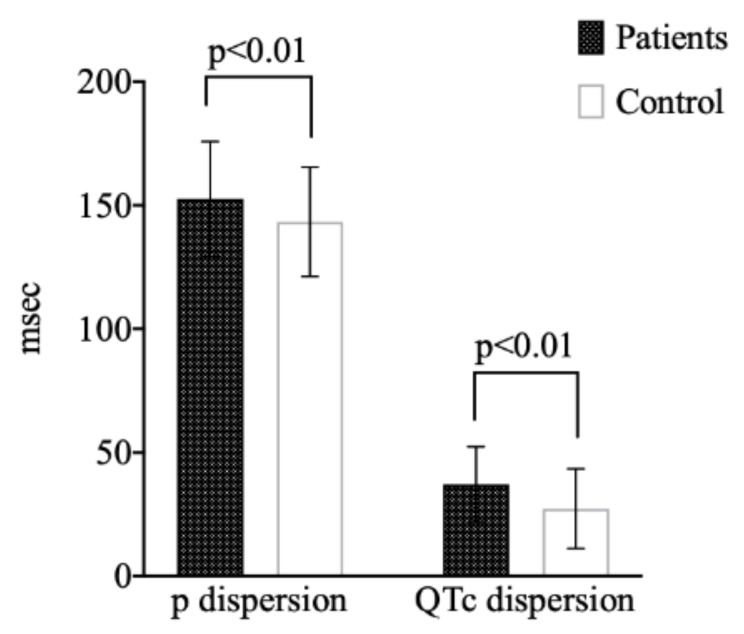
QTc dispersion and p dispersion values between groups. (QTc, corrected QT; QT, defines the period of ventricular repolarization).

There was no significant difference between groups in terms of age, body mass index, gender, blood pressures, biochemical and hematological analyzes, and echocardiographic evaluation (*p* > 0.05). Heart rate, RR interval, P-min, QT-min, QT-max, QTc-min, and QTc-max were similar between patient and control groups (*p* > 0.05). QTd, QTcd, and Pd values were significantly higher in patients than in the healthy individuals (*p* < 0.01). Figure 1 presents the QTcd and Pd differences between patient and control groups.

The clinical and demographic characteristics of the patients using carbamazepine, VPA, and control groups are presented in Table 2. 

The clinical and demographic data were similar in study groups except for ECG analysis. QTd, QTcd, and Pd values were significantly higher in patients using carbamazepine and VPA than in control subjects (*p* < 0.01). QTcd and Pd values were similar in patients using carbamazepine and VPA (*p* > 0.05). 

**Figure 2:**
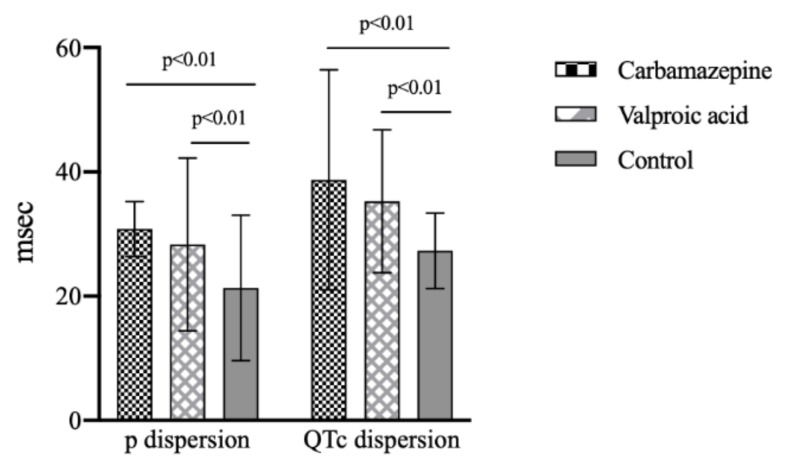
QTc dispersion and p dispersion values between drug and control groups. (QTc, corrected QT).

QTcd and Pd values between carbamazepine, VPA, and control groups are shown in Figure 2. 

Table 3 shows the ECG analysis between groups according to gender.

Female patients had significantly higher QTcd and Pd values compared to healthy female subjects (QTcd, *p* = 0.01; Pd, *p* = 0.01). QTcd, Pd, and P-max (P-maximum) values were statistically higher in male patients compared to healthy male controls (QTcd, *p* = 0.01; Pd, *p* = 0.01; P-max, *p* < 0.01). You can find the result of ECG analysis on carbamazepine, VPA, and control groups in terms of gender in Table 4. 

**Figure 3:**
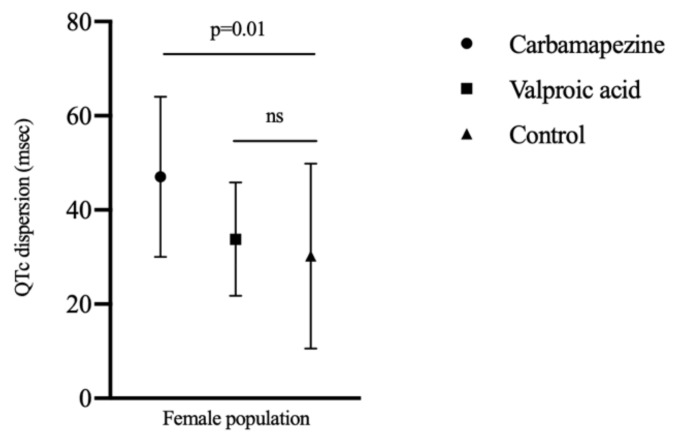
QTc dispersion values between groups according to female population. (QTc, corrected QT; ns, nonsignificant).

P-min, P-max, QTc-min, and QTc-max values were similar in female subjects in carbamazepine, VPA, and control groups (*p* > 0.05). QTcd values were significantly higher in female patients using carbamazepine compared to female population in VPA and control groups (*p* = 0.01). Figure 3 shows the QTcd values in female population with carbamazepine, VPA, and control groups. 

**Figure 4:**
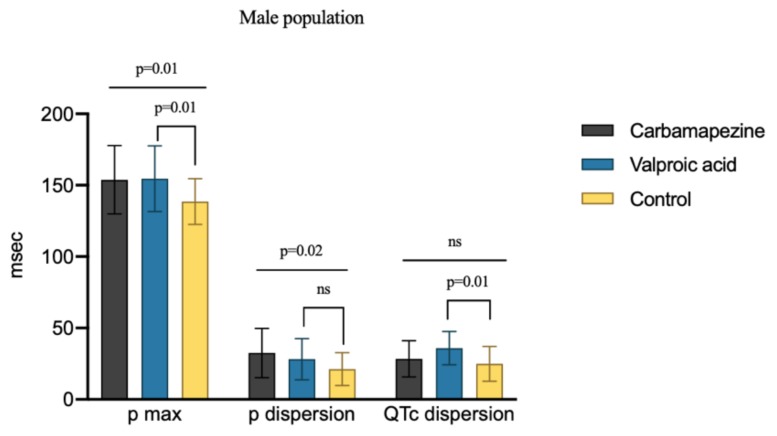
QTc, p dispersion, and p maximum values between drug and control groups, according to male population. (QTc, corrected QT; ns, nonsignificant).

In the male population, P-min, QTc-min, and QTc-max values were similar in carbamazepine, VPA, and control groups (*p* > 0.05). Pd values were significantly higher in male patients using carbamazepine compared to male epilepsy patients on VPA and healthy controls (*p* = 0.02). Male patients using VPA had significantly higher QTcd values compared to male epilepsy patients on carbamazepine and controls (*p* = 0.01). Figure 4 presents the QTcd, Pd, and P-max values in the male population with carbamazepine, VPA, and control groups.

Table 5 shows the ECG analysis between male and female patients.

## 4. Discussion

In our study, we demonstrated five main results in patients with GTCS. Our first result was significantly higher QTd, QTcd, and Pd values in epilepsy patients compared to healthy controls. Our second result was that female patients using carbamazepine had higher QTcd values compared to female patients on VPA and control groups. Third, male epilepsy patients using carbamazepine had higher Pd values compared to male epilepsy patients on VPA and control groups. Four, female patients had higher QTcd and QTd values compared to male patients. The last but not least finding was the significantly higher QTcd values in male patients on VPA group compared to male epilepsy patients on carbamazepine and control groups. Dogan et al. have evaluated depolarization indices in patients with partial epilepsy and found significantly longer QTc-max and QTcd intervals in patients with epilepsy compared to controls [16]. Moreover, they indicated that there was no difference in QT intervals between patients treated with carbamazepine and non-carbamazepine antiepileptic drugs. 

QT interval is an indicator of the period of ventricular depolarization and repolarization. A prolonged QT interval and QTd are defined as cardiac repolarization abnormalities and they are relate to sudden cardiac death and ventricular tachyarrhythmia [17]. The P-wave duration shows the contraction and depolarization of atrial muscle. Pd represents the homogeneity of inter- and intra-atrial conduction [18]. Souza et al. observed prolonged P-wave in patients with epilepsy than in controls [19]. In epilepsy patients, it was shown that cardiac repolarization abnormalities may cause SUDEP due to ventricular tachyarrhythmia. A possible explanation might be altered cardiac ion channel expression due to seizure activity which leads to cardiac dysfunction. [20] The underlying etiology of QTd is unclear in patients with epilepsy. Autonomic dysfunction with non-uniform cardiac sympathetic innervation and increased sympathetic tone have been shown to be related to epilepsy which may cause prolonged QTd [21]. The prolongation of QTd and QTcd values indicate that epilepsy patients had ventricular repolarization abnormalities. Also, these findings show the possibility of development of ventricular arrhythmia and SUDEP in the interictal period in epilepsy patients. 

AEDs can facilitate the development of ventricular tachyarrhythmias by acting on ionic channels. AEDs may pose a pro-arrhythmic effect if used in combination [22]. Carbamazepine blocks the sodium channels in the cardiac conduction system, and it may cause an atrioventricular block, ventricular dysrhythmia, and syncope [23]. There are conflicting results related to carbamazepine in literature. A study conducted by Matteoli et al. showed that carbamazepine therapy does not cause QT interval prolongation [24]. Amin et al. found that carbamazepine mono-therapy was not related to QTc interval prolongation in generalized epilepsy patients [13]. A clinical trial in newly diagnosed epilepsy patients showed that carbamazepine did not cause any significant changes in QTc interval [25]. However, there are studies claiming that carbamazepine use increases the risk of SUDEP [26,27,28]. It is well known that VPA possesses an antiarrhythmic action. Surges et al. demonstrated the effect of valproate on QTc prolongation [29]. Amin et al. showed that VPA reduced the number of atrial ectopic in patients with GTCS [13]. 

In addition, we evaluated epilepsy patients in terms of gender. We found that female patients with epilepsy were more prone to ventricular arrhythmias compared to healthy female individuals. Male epilepsy patients were prone to ventricular repolarization and atrial depolarization abnormalities compared to healthy male controls. Also, we evaluated repolarization indices in epilepsy patients in terms of carbamazepine and VPA usage. QTcd values in female patients using carbamazepine were significantly higher compared to the female epilepsy patients on VPA and control groups. Male patients using VPA had significantly higher QTcd values than the male epilepsy patients on carbamazepine and control groups. We concluded that female patients using carbamazepine and male patients using VPA were prone to ventricular arrhythmia compared to the control group. The possible influences of VPA use on cardiac conduction, duration of the epilepsy, VPA dosage, and the influences of the epilepsy disorder itself are also important factors, which would be responsible for some higher repolarization parameters in VPA group. Studies with more patients and multicenters are necessary to confirm these results. 

### Limitations

This study has some limitations. It was a single-center study enrolling a relatively small group of patients, and our results cannot be generalized to all health centers. Another study limitation was the single measurement of repolarization indices, and these parameters might change in the course of time, especially with AEDs. One other limitation of this study was the lack of information on carbamazepine and VPA dosages. Moreover, patients with other AEDs were not investigated in the study. In addition, we did not evaluate peri-ictal ECG parameters and different epilepsy types in the study. Despite these limitations, our study provides important information on the repolarization indices in epilepsy patients.

## 5. Conclusions

In conclusion, we determined that QTd, QTcd, and Pd values were significantly higher in epileptic patients than in healthy controls. Also, we showed that female patients using carbamazepine and male patients using VPA were prone to ventricular arrhythmia compared to the control group. More studies are necessary to confirm these results. 

## Figures and Tables

**Table 1:** The demographic and clinical information of participants.

	Patients (*n* = 66)	Control (*n* = 63)	*p*
Age (years) (Mean±SD)	29.4 ± 12.2	33.3 ± 12.1	0.07
Male/Female	38/28	34/29	0.68
BMI	30.1 ± 3.9	28.5 ± 4.7	0.06
Systolic blood pressure (mmHg)	123.9 ± 12.9	124.0 ± 11.4	0.96
Diastolic blood pressure (mmHg)	77.7 ± 7.1	77.5 ± 6.7	0.87
Ejection fraction (%)	63.8 ± 2.5	63.8 ± 2.7	0.99
LVEDD (cm)	4.1 ± 0.2	4.0 ± 0.3	0.98
LVESD (cm)	2.5 ± 0.3	2.6 ± 0.2	0.53
Left atrial diameter (mm)	3.2 ± 0.4	3.1 ± 0.3	0.63
Mitral E (m/sec)	0.8 ± 0.1	0.7 ± 0.1	0.64
Mitral A (m/sec)	0.6 ± 0.2	0.6 ± 0.1	0.36
IVS (cm)	0.9 ± 0.1	0.8 ± 0.1	0.84
PWD (cm)	0.9 ± 0.2	0.9 ± 0.1	0.50
Aorta systolic diameter (mm)	2.6 ± 0.2	2.5 ± 0.3	0.97
Aorta diastolic diameter (mm)	2.8 ± 0.3	2.7 ± 0.2	0.80
Heart rate (bpm)	79.2 ± 9.2	78.4 ± 9.8	0.65
P max (msec)	152.6 ± 23.2	143.3 ± 22.1	0.02
P min (msec)	122.9 ± 18.5	122.1 ± 19.0	0.81
P dispersion (msec)	29.7 ± 14.1	21.3 ± 11.7	<0.01
R-R interval (msec)	767.6 ± 90.4	776.5 ± 98.4	0.59
QT max (msec)	422.7 ± 40.3	414.1 ± 42.6	0.23
QT min (msec)	390.2 ± 40.4	389.9 ± 39.9	0.96
QT dispersion (msec)	32.6 ± 13.5	24.3 ± 14.7	0.001
QTc max (msec)	483.5 ± 39.8	470.8 ± 36.2	0.06
QTc min (msec)	446.4 ± 41.3	443.2 ± 34.7	0.64
QTc dispersion (msec)	37.2 ± 15.2	27.3 ± 16.1	<0.01
Duration of epilepsy (year)	12.5 ± 4.5	–	

Note: BMI, body mass index; LVEDD, left ventricular end-diastolic diameter; LVESD, left ventricular end-systolic diameter; mitral E, early diastolic mitral flow velocity; mitral A, late diastolic mitral flow velocity; IVS, interventricular septum; PWD, posterior wall diameter; p max, p maximum; p min, p minimum.

**Table 2:** The data of the drug groups and control groups.

	Carbamazepine (*n* = 36)	Valproic Acid (*n* = 30)	Control (*n* = 60)	*p*
Age (years)	30.4 ± 13.4	28.3 ± 10.5	33.3 ± 12.1	0.15
Male n (%)	16 (44)	22 (73)	34 (54)	0.05
BMI	29.9 ± 3.8	30.3 ± 4.1	28.5 ± 4.7	0.11
Systolic blood pressure (mmHg)	126.3 ± 11.1	121.2 ± 14.5	124.0 ± 11.4	0.24
Diastolic blood pressure (mmHg)	78.1 ± 6.8	77.2 ± 7.5	77.5 ± 6.7	0.86
Ejection Fraction (%)	63.5 ± 2.3	64.2 ± 2.8	63.8 ± 2.7	0.5
LVEDD (cm)	4.0 ± 0.3	4.0 ± 0.3	4.0 ± 0.3	0.91
LVESD (cm)	2.4 ± 0.3	2.5 ± 0.2	2.6 ± 0.2	0.81
Left atrial diameter (mm)	3.1 ± 0.3	3.1 ± 0.2	3.1 ± 0.3	0.89
Mitral E (m/sec)	0.7 ± 0.1	0.6 ± 0.1	0.7 ± 0.1	0.37
Mitral A (m/sec)	0.6 ± 0.1	0.5 ± 0.1	0.6 ± 0.1	0.35
IVS (cm)	0.9 ± 0.1	1.0 ± 0.1	0.8 ± 0.1	0.72
PWD (cm)	0.9 ± 0.1	0.8 ± 0.1	0.9 ± 0.1	0.67
Aorta systolic diameter (mm)	2.5 ± 0.2	2.5 ± 0.2	2.5 ± 0.3	0.92
Aorta diastolic diameter (mm)	2.8 ± 0.2	2.7 ± 0.2	2.7 ± 0.2	0.85
Heart rate (bpm)	78.6 ± 8.7	79.9 ± 9.8	78.4 ± 9.8	0.76
P max (msec)	153.9 ± 23.7	151.0 ± 22.9	143.3 ± 22.1	0.06
P min (msec)	123.1 ± 18.0	122.7 ± 19.5	122.1 ± 19.0	0.97
P dispersion (msec)	30.8 ± 14.4 *	28.3 ± 13.9 *	21.3 ± 11.7	<0.01
R-R interval (msec)	772.5 ± 84.7	761.7 ± 98.0	776.5 ± 98.4	0.78
QT max (msec)	428.5 ± 45.3	415.5 ± 32.6	414.1 ± 42.6	0.21
QT min (msec)	394.8 ± 44.6	384.8 ± 34.8	389.9 ± 39.9	0.60
QT dispersion (msec)	34.1 ± 15.7 *	30.8 ± 10.1 *	24.3 ± 14.7	0.003
QTc max (msec)	488.8 ± 47.4	477.3 ± 27.7	470.8 ± 36.2	0.08
QTc min (msec)	450.0 ± 47.8	442.0 ± 32.1	443.2 ± 34.7	0.63
QTc dispersion (msec)	38.7 ± 17.7 *	35.3 ± 11.5 *	27.3 ± 16.1	<0.01

Note: BMI, body mass index; LVEDD, left ventricular end-diastolic diameter; LVESD, left ventricular end-systolic diameter; mitral E, early diastolic mitral flow velocity; mitral A, late diastolic mitral flow velocity; IVS, interventricular septum; PWD, posterior wall diameter; p max, p maximum; p min, p minimum. * Control group versus other study groups.

**Table 3:** Electrocardiographic data of patient and control groups, in terms of gender.

Female		Patients	Control	*p*
	P max (msec)	150.4 ± 23.6	149.0 ± 26.8	0.84
	P min (msec)	121.1 ± 18.3	127.6 ± 23.7	0.25
	P dispersion (msec)	29.3 ± 12.1	21.4 ± 12.2	0.02
	QTc max (msec)	494.6 ± 48.6	476.9 ± 43.5	0.15
	QTc min (msec)	451.8 ± 51.3	446.6 ± 42.0	0.68
	QTc dispersion (msec)	43.2 ± 16.7	30.2 ± 19.6	0.01
Male				
	P max (msec)	154.2 ± 23.1	138.5 ± 16.0	<0.01
	P min (msec)	124.2 ± 18.8	117.4 ± 12.4	0.08
	P dispersion (msec)	30.0 ± 15.6	21.2 ± 11.5	0.01
	QTc max (msec)	475.4 ± 30.1	465.5 ± 28.2	0.16
	QTc min (msec)	442.4 ± 32.2	440.4 ± 27.4	0.77
	QTc dispersion (msec)	32.7 ± 12.5	24.9 ± 12.1	0.01

**Table 4:** Electrocardiography (ECG) analysis results on carbamazepine, valproic acid, and control groups, in terms of gender.

Female		Carbamazepine	Valproic Acid	Control	*p*
	P max (msec)	154.0 ± 24.1	141.3 ± 21.0	149.0 ± 26.8	0.48
	P min (msec)	124.5 ± 19.6	112.5 ± 11.6	127.6 ± 23.7	0.21
	P dispersion (msec)	29.5 ± 11.9	28.8 ± 13.6	21.4 ± 12.2	0.06
	QTc max (msec)	496.7 ± 52.3	489.5 ± 40.3	476.9 ± 43.5	0.34
	QTc min (msec)	450.2 ± 54.3	455.8 ± 46.2	446.6 ± 42.0	0.88
	QTc dispersion (msec)	47.0 ± 17.0 *	33.8 ± 12.0	30.2 ± 19.6	0.01
Male					
	P max (msec)	153.8 ± 23.9 *	154.5 ± 23.0 *	138.5 ± 16.0	0.01
	P min (msec)	121.3 ± 16.3	126.4 ± 20.6	117.4 ± 12.4	0.13
	P dispersion (msec)	32.5 ± 17.3 *	28.2 ± 14.4	21.2 ± 11.5	0.02
	QTc max (msec)	478.9 ± 40.0	472.9 ± 21.0	465.5 ± 28.2	0.30
	QTc min (msec)	449.9 ± 40.0	437.0 ± 24.7	440.4 ± 27.4	0.41
	QTc dispersion (msec)	28.4 ± 12.7	35.9 ± 11.6 *	24.9 ± 12.1	0.01

* Control group versus other study groups.

**Table 5:** Electrocardiographic data of patients, in terms of gender.

	Female (*n* = 28)	Male (*n* = 38)	*p*
p max	150.4 ± 23.6	154.2 ± 23.1	0.51
p min	121.1 ± 18.3	124.2 ± 18.8	0.50
p dispersion	29.3 ± 12.1	30.0 ± 15.6	0.84
QTc max	494.6 ± 48.6	475.4 ± 30.1	0.07
QTc min	451.8 ± 51.3	442.4 ± 32.2	0.37
QTc dispersion	43.2 ± 16.7	32.7 ± 12.5	<0.01
QT max	432.0 ± 42.4	415.9 ± 37.7	0.11
QT min	394.6 ± 43.8	387.0 ± 38.1	0.46
QT dispersion	37.5 ± 14.9	28.9 ± 11.2	0.01

Note: QTc, corrected QT, QTc max, QTc min, QT max, QT min, p max, p min, and p dispersion values were similar in male and female patients (*p* > 0.05). Female patients had significantly higher QTcd and QTd values compared to male patients (QTcd, *p* < 0.01; QTd, *p* = 0.01).
